# Intratympanic gentamicin treatment for Ménière’s disease: a randomized, double-blind, placebo-controlled trial on dose efficacy - results of a prematurely ended study

**DOI:** 10.1186/1745-6215-15-328

**Published:** 2014-08-18

**Authors:** Hendrik G Bremer, Ingrid van Rooy, Bas Pullens, Carla Colijn, Inge Stegeman, Hester J van der Zaag-Loonen, Peter Paul van Benthem, Sjaak F L Klis, Wilko Grolman, Tjasse D Bruintjes

**Affiliations:** Department of Otorhinolaryngology, University Medical Center Utrecht, Heidelberglaan 100, Room G.02.531, Utrecht, 3584 CX The Netherlands; Brain Center Rudolf Magnus, University Medical Center, Utrecht, The Netherlands; Department of Otorhinolaryngology, Gelre Ziekenhuizen Apeldoorn, Apeldoorn, The Netherlands; Department of Biometry, Gelre Ziekenhuizen Apeldoorn, Apeldoorn, The Netherlands; Department of Otorhinolaryngology, Erasmus Medical Center/ Sophia Children’s Hospital, Rotterdam, The Netherlands

**Keywords:** Aminoglycosides, Gentamicin, Intratympanic, Ménière’s disease, DHI, Audiometry, RCT, Double-blind design, Adverse event

## Abstract

**Background:**

Gentamicin is used as a therapeutic agent for Ménière’s disease because of its vestibulotoxicity causing chemo-ablation of the vestibular sensory epithelia. Its use has increased in recent years. However, there is still no consensus about the dose regimen of gentamicin in the treatment of Ménière’s disease. In this study two different dose regimen treatment protocols are compared in a placebo controlled study design. The primary objective is to quantify the treatment effect on dizziness, the secondary objective is hearing evaluation.

**Methods:**

We performed a randomized, double-blind, placebo-controlled study in adults with unilateral Ménière’s disease according to the AAO-HNS guidelines resistant to conservative medication. Three groups received four injections, administered weekly (four intratympanic injections with 40 mg/mL gentamicin solution, two injections gentamicin solution and two injections of placebo in random order, or four injections with placebo). Outcome measures were the score on the Dizziness Handicap Inventory and pure tone audiometry (PTA). Intended follow-up was 2 years.

**Results:**

During follow-up one patient exceeded the accepted amount of hearing loss. Further, enrollment was very slow (until 12 months between two patients) and new insights showed an apparent benefit of intratympanic gentamicin treatment (ITG). Therefore we performed an unscheduled interim analysis which showed that PTA threshold shifts reached the stopping criteria in two more patients. Because of this, this study was ended. Of the three patients with the significant PTA threshold shift two were enrolled in the gentamicin group.

**Conclusion:**

No conclusions can be drawn concerning doses regimens. Now that new publications have shown that ITG treatment can be an effective and safe treatment, a placebo-controlled randomized controlled trial may not pass the ethical committee because of these recent reports in literature. Still, a dose regimen study (without placebo) on ITG treatment needs to be performed.

**Trial registration:**

This trial was registered in The University Medical Center Utrecht/ Gelre hospital Apeldoorn. Protocol ID: 07/343, EudraCT number 2006-005913-37.

## Background

Ménière’s disease (MD) is an invalidating illness characterized by attacks of vertigo with hearing loss, tinnitus, and/or aural fullness of the affected ear. The exact origin of this disease is not known, but it is generally accepted that endolymphatic hydrops is the pathophysiological substrate. Treatment, besides advises on living habits and diet, exists of medication. This treatment gives unsatisfying results in many cases. The natural course of this disease often results in a decline of the sensitivity of the vestibular peripheral system and progressive hearing loss [1]. Gentamicin is an aminoglycoside antibiotic that is applied systemically for serious infections. Some of the adverse events of gentamicin treatment are vestibulotoxicity and cochleotoxicity. A common feature of aminoglycoside ototoxicity is that delayed hearing impairment can occur after cessation of treatment. This delay is affected by different factors and takes place at multiple levels. Aminoglycosides enter the cochlea rapidly following administration (systemic and intratympanic) and reach a plateau within 0.5 to 3 h after application. Clearance from peri- and endolymph is slow and aminoglycosides persist in the inner ear fluids and tissues long after the drug has been removed from the blood by renal clearance. The drugs may persist in the inner ear fluids for months after treatment and this may account for delayed hair cell degeneration.

For several reasons gentamicin is more vestibulotoxic than cochleotoxic [2]. Intratympanic gentamicin treatment (ITG) for control of vertigo has become popular worldwide. In this treatment gentamicin solution is applied in the middle ear with the aim of damaging the vestibular hair cells with an intended result of a decrease (or disappearing) of vertigo complaints while preserving hearing [1, 3–6].

Several studies have been published about this topic. These are mainly descriptive studies that used different gentamicin concentrations and administration protocols. Unambiguous in these publications is the high success rate in reducing the number of vertigo attacks. However, still obscure is the best treatment scheme and optimal concentration of gentamicin. Two dose regimen protocols are mostly used. One model uses a fixed number of administrations (fixed dose regimen) and the other model uses a titration regimen where gentamicin is administered until attacks disappeared or other terms are reached [3, 7–13]. Different papers, including two meta-analyses, showed that the degree in dizziness control is not related to the amount of vestibular functional loss after ITG treatment [7, 10, 11]. There are to date a few placebo-controlled studies on this topic available; for example, the study by Stokroos et al. (2004) [14] used a titration model and the study by Postema et al. (2008) [15] used a fixed dose regimen. Although the studies used a different treatment protocol, both studies demonstrated the efficacy of gentamicin treatment for Ménière’s disease when compared to placebo treatment. A weakness of these studies is the limited follow-up time. Stokroos et al. used a follow-up period of 6 months and Postema et al. of 12 months. The advice of the Committee on Hearing and Equilibrium of the American Association of Otolaryngology - Head and Neck Surgery (1995) is to use a follow-up of at least 24 months, taking into consideration the expected placebo effects (Committee on Hearing and Disequilibrium, 1995). In this study we compare the efficacy of two different administration protocols. One uses four injections with gentamicin solution (40 mg/mL) *versus* two injections with gentamicin solution (40 mg/mL), both compared to placebo treatment. The main question is whether ablation of the peripheral vestibular system (four injections) gives a better clinical result than more subtle damage to the peripheral vestibular system (two injections). Secondary outcome is the effect of treatment on hearing. Numbers of injections are based on the studies by Stokroos et al. (1.5 +/- 0.5 injections) and Postema et al. (4 weekly injections). The fact that to date no placebo-controlled dose efficacy study is available is an extra argument for a placebo group. We expect a better clinical result after four injections with gentamicin solution.

## Methods

The study was started in two hospitals, the University Medical Center in Utrecht (UMC Utrecht) and Gelre Hospital Apeldoorn. Before entering the study all patients gave written informed consent for participation in the study. Patients were eligible for the study if they had been diagnosed as having unilateral Ménière’s disease according to the 1995 AAO-HNS criteria: two or more spontaneous episodes of vertigo each lasting 20 min or longer, sensorineural hearing loss documented audiometrically in the diseased ear and the presence of tinnitus, and/or aural fullness in this ear. Causes other than Ménière’s disease were excluded by a diagnostic protocol, including vestibular tests, MRI of the cerebellopontine angle, clinical history, and physical examination. Other inclusion criteria were: Ménière’s disease resistant to conservative medical treatment executed longer than 6 months, (that is, Dizziness Handicap Score of at least 30 points) and ability to provide written informed consent. Patients had to have compromised hearing on the affected side without fluctuations. Exclusion criteria were ipsilateral middle ear pathology, contralateral ear pathology or contralateral hearing loss, allergy for aminoglycosides, or earlier treatment with intratympanic gentamicin. After ITG treatment patients were advised to visit the physiotherapist for, for example, Cawthorn-Cooksey exercises or other patient-specific exercises. Approval of the study was granted by the medical ethics testing committee (METC) of both the University Medical Center Utrecht and Gelre Hospitals Apeldoorn (on 30 December 2008, Protocol ID: 07/343, EudraCT number 2006-005913-37).

For assignment of the participants a computer-generated list of random numbers was used. Patients were randomized to one of three treatment groups. The three groups all received four weekly intratympanic injections. Group 1 received placebo injections (sterile NaCl 0.9% solution), group 2 received two injections with gentamicin 40 mg/mL and two injections with placebo in random order, and group 3 received four injections with gentamicin 40 mg/mL. After treatment the intended follow-up period was 2 years. Three-monthly questionnaires were taken by telephone. If there was any problem the patients were free to contact one of the researchers and all the time they were free to withdraw from the study. At the end of the follow-up a last audiogram, ENG, and the DHI questionnaire were taken.

Primary outcome parameter was the total score on the Dizziness Handicap Inventory (DHI). The DHI is a standardized and validated questionnaire assessing impairments due to dizziness [16–18]. It comprises 25 items, leading to a score range from 0 to 100, with higher scores indicating more perceived impairments. The secondary outcome parameter was hearing loss. Intended follow-up was 2 years. Stopping criteria were the occurrence of SUSARs (Suspected Unexpected Serious Adverse Reactions), one of which was an average hearing deterioration of 30 dB or more over the frequencies of 500, 1,000, 2,000, and 4,000 Hz of the treated ear, or an average deterioration of 15 dB or more over the frequencies of 500, 1,000, 2,000, and 4,000 Hz at the contralateral ear. Harms were reported following the CONSORT extension for harms. During follow-up at the outpatient clinic, the audiograms of the patients were visually compared with the former audiograms.

### Statistical analysis

Power analysis (alfa at 5% and power at 80%) showed that 16 patients per group were needed to show a clinically relevant difference of 12 points on the DHI scores between the three groups [19]. The first interim analysis was planned to be performed when 18 patients were included.

DHI scores and hearing outcomes were compared between the three groups with non-parametric statistics, due to the small numbers (Kruskal Wallis test for continuous variables).

## Results

Between June 2009 and January 2012 we included 15 patients. One patient was included in the UMC Utrecht and 14 in Gelre Hospital. The time between inclusion of the 14th and the 15th patient was 12 months. Mean age was 64.8 years (SD 12.5 years), eight (57%) were men. Five (one man) were randomized in group 1, five (four men) in group 2, and five (three men) in group 3 (Table 1). However, one patient (group 2) withdrew from the study after randomization. Although informed consent was obtained before inclusion, she changed her mind and did not want the chance to be treated with placebo and was not included in the further follow-up and analyses. Another patient withdrew from the study after two injections because he suffered from Tumarkins crises. One patient died during the study (as a result of co-morbidity). This patient was enrolled in the gentamicin-placebo group (group 2). The median duration in years of MD was 2.5 (range, 0.1 to 18.2) for the placebo-group, 3.3 (range, 0.7 to 7.5) for the placebo-gentamicin group, and 3.1 (range, 1.1 to 19.6) for the gentamicin group. Because of the slow enrollment, apparent hearing loss in one patient and new insights with respect to the benefit of ITG treatment the randomization code was broken and we performed an unscheduled interim analysis on which the following results are based.

**Table 1:** **Demographic, clinical, and treatment characteristics of included patients**

Characteristics	Total	Placebo	Gentamicin/Placebo	Gentamicin
	n = 15	n = 5	n = 4	n = 5
Sex				
Male	8 (53%)	1 (20%)	4 (100%)	3 (60%)
Age (mean, (SD))	64.8 (12.5)	57.3 (16.7)	72.6 (5)	64.5 (8)
Duration of MD in years (median (range))	3 (0.1-19.6)	2.5 (0.1-18.2)	3.3 (0.7-7.5)	3.1 (1.1-19.6)
DHI	48.6 (20.0)	54.0 (18.0)	40.5 (16.4)	51.2 (25.5)
Averaged (0.5, 1, 2, 4 kHz) pure-tone-hearing loss (dB HL)				
Right	41.2 (19.9)	35.0 (24.5)	29.7 (7.4)	59.0 (8.6)
Left	50.6 (23.7)	43.8 (21.6)	61.3 (30.7)	46.8 (21.3)

### Effect of treatment: vertigo

The median DHI score before treatment was 54 points (range, 32 to 76), 44 points (range, 18 to 56), and 46 points (range, 20 to 88) for the placebo group, the gentamicin-placebo group, and the gentamicin group, respectively. At the time of the interim analysis the median DHI score for the placebo group was 24 points (range, 4 to 34, decrease of 20 points), for the gentamicin-placebo group 34 points (range, 4 to 58, decrease of 10 points), and for the gentamicin group 5 points (range, 0 to 76, decrease of 41 points); the latter group showed the largest decrease in DHI score but this was not statistically significant (*P* >0.5).

### Side-effect of treatment: hearing outcome

During follow-up, one patient had a hearing loss of 50 dB of the treated side averaged over the four frequencies which was more than the criterium of 30 dB. The interim analysis showed two more patients with a hearing loss of more than 30 dB. Both had an exceeding of the accepted threshold shift (30 dB) of approximately 5 dB. After breaking the randomization code, the patient with the PTA shift of 50 dB was enrolled in the gentamicin group. Of the patients with the minor exceeding, one was also enrolled in the gentamicin group and the other patient was enrolled in the placebo group. The mean increase in PTA threshold shift for the placebo group was 10 60;dB, for the gentamicin-placebo group 0.9 dB and for the gentamicin group 27.4 dB (*P* >0.5).

## Discussion

The present study was performed to assess a good dose regimen protocol for ITG therapy in patients suffering from Ménière’s disease. Unfortunately, no conclusive statements can be made about this due to the fact that the study was ended prematurely and at that time too few patients have been included to make significant conclusions. However, our results indicate that there does seem to be efficacy of gentamicin treatment in comparison to placebo but the significant risk for hearing loss with four injections enervates these results.

The time between inclusion of the 14th patient and 15th patient was 12 months indicating a very slow enrolment. During that time a Cochrane Review was published which stated that ITG treatment was an effective and safe therapy for patients with Ménière’s disease [6]. Also, a meta-analysis showed an effective and safe treatment with intratympanic steroids [20]. Data monitoring committees have an ethical obligation to ensure that patients are offered effective treatment as soon as it is clear that an effective treatment is indeed available [21]. Based on the slow enrollment, the suspicion of more hearing loss besides the noticed exceeding in PTA shift of one patient and together with the new scientific insights we decided to perform the unscheduled interim analysis over the 15 patients that had been included in our study until that time. The results of this interim analysis showed two other (minor) exceeding in PTA shift. This meant that the stopping criterion was reached and the study was ended. Because of this unexpected stop of the study full inclusion was not reached resulting in too small a sample size to support a strong external validity of our study. Clinicians should consider these results with caution [22].

Retrospectively, visual determination of the audiograms was not sufficient because two of the three exceedings of the criteria for hearing loss were only noticed after the unscheduled interim analysis. If, for example, the time courses of hearing loss are calculated during outpatient clinic visits, also a small exceeding in accepted hearing loss is noticed. Two of the three patients were enrolled in the gentamicin group so the PTA threshold shifts were probably the result of the cochleotoxicity of gentamicin. The third patient was enrolled in the placebo group so in this case the MD itself instead of the ototoxicity of gentamicin may have caused the PTA threshold shift; the natural course of Ménière’s disease eventually leads to a sensorineural hearing loss of approximately 50 dB [1]. The two patients of the gentamicin group are content with their treatment as indicated by a strong reduced DHI score. One other patient suffered from residual Ménière’s attacks with Tumarkins crises after four injections and withdrew from the study. This patient was enrolled in the placebo group, so the cause of the crises was MD itself and not gentamicin treatment. Subsequently, this patient received one injection with gentamicin in the outpatient clinic (outside the scope of this study) with satisfying results. We assessed the stopping criteria for hearing loss for the PTA threshold shift at 30 dB averaged over the four frequencies. Maybe some MD patients will accept the chance of hearing loss as they are eager to be treated for their main problem, that is, disabling vertigo attacks. So maybe the determined stopping criterion for hearing loss can be augmented in future studies.

The difficulty of recruiting enough Ménière subjects for a 2-year follow-up period with adherence to the 1995 AAO-HNS guidelines for reporting is well known. There is also a certain placebo effect when treating patients with MD. This was the reason that this RCT contained a placebo group. Frequently, this discouraged patients to participate in the trial since they did not want to receive placebo instead of gentamicin. These patients suffered from recalcitrant MD, with a great morbidity, and they demanded a ‘real’ treatment and did not accept the risk to get treated with placebo. For this reason, studies have been performed where two treatment modalities (for example, intratympanic gentamicin *vs.* dexamethason or prednison) are being compared [23, 24]. The latter study implicated less efficacy of prednison compared to ITG injections without a difference in hearing level. A low-dose protocol can be used [25–27], administrating just one or two gentamicin injections with a similar effectiveness for vertigo control and with a lower risk for major side-effects (that is, hearing loss and a prolonged period of imbalance after treatment) as compared with high-dose protocol [10]. But there are limitations on the latter study regarding the variability in the total dosage of gentamicin delivered (2.4 to 720 mg). Wasson et al. [28] indicated efficacy of ITG also in the longer term (mean, 17 years and 3 months).

## Conclusion

The known inherent risk of gentamicin treatment induced hearing loss was also present in our study. Due to a slow enrollment of patients into our study and recent new clinical insights into the role and treatment benefits of ITG in MD patients we terminated our study prematurely. We performed an interim analysis that revealed a potential SUSAR that we reported to our Medical Ethical Committee. This interim analysis did show some efficacy of four injections of gentamicin compared with placebo. Important consideration beside its potential benefit is the risk to perceptive hearing loss. To adapt a placebo controlled study design, now that the reports in literature show effectiveness for ITG seems unethical. [6, 20, 28]. However, a randomized controlled trial on dose regimens still has to be performed because of the lack of consensus on the intratympanic dose of gentamicin that needs to be used to have the best balanced result between vertigo reduction and hearing preservation.

### Trial status

At the time of submission, 31% of the participants have been included in the trial.

## Abbreviations

AAO-HNS: American Academy of Otolaryngology - Head and Neck Surgery
dB: Decibel
DHI: Dizziness Handicap Inventory
ENG: Electronystogram
MD: Ménière’s disease
NaCl: Natrium chloride
PTA: Pure tone audiometry
RCT: Randomized controlled trial
SD: Standard deviation
SUSARs: Suspected unexpected serious adverse reactions
UMCU: University Medical Center Utrecht.
